# European starlings unriddle the ambiguous-cue problem

**DOI:** 10.3389/fpsyg.2014.00944

**Published:** 2014-08-26

**Authors:** Marco Vasconcelos, Tiago Monteiro

**Affiliations:** ^1^Animal Learning and Behavior Lab, School of Psychology, University of MinhoBraga, Portugal; ^2^Behavioral Ecology Research Group, Department of Zoology, University of OxfordOxford, UK; ^3^Champalimaud Neuroscience Program, Champalimaud Centre for the UnknownLisbon, Portugal

**Keywords:** ambiguous-cue problem, configural cues, interfering cue hypothesis, partial reinforcement, starlings, value transfer theory

## Abstract

The ambiguous-cue problem is deceptively simple. It involves two concurrently trained simultaneous discriminations (known as PA and NA trials), but only three stimuli. Stimulus A is common to both discriminations, but serves as non-reinforced stimulus (S-) on PA trials and as reinforced stimulus (S+) on NA trials. Typically, animals’ accuracy is lower on PA trials—the ambiguous-cue effect. We conducted two experiments with European starlings (*Sturnus vulgaris*) using Urcuioli and Michalek’s (2007, Psychon B Rev 14, 658–662) experimental manipulations as a springboard to test the predictions of two of the most important theoretical accounts of the effect: the interfering cue hypothesis and value transfer theory. Both experiments included two groups of birds, one trained with a regular ambiguous-cue problem (Group Continuous) and another trained with partial reinforcement on PA trials (Group PA-Partial). The experiments differed only in the number of sessions (18 vs. 36) and daily trials (360 vs. 60). As previously observed, we found faster acquisition on NA trials than on PA trials in both experiments, but by the end of training PA performance was surprisingly high, such that no ambiguous-cue effect was present in Group Continuous of either experiment. The effect was still present in both PA-Partial groups, but to a smaller degree than expected. These findings are inconsistent with the literature, in particular with the results of Urcuioli and Michalek (2007) with pigeons, and question the aforementioned theoretical accounts as complete explanations of the ambiguous-cue effect. In our view, to achieve such high levels of accuracy on PA trials, starlings must have attended to configural (i.e., contextual) cues, thus differentiating stimulus A when presented on PA trials from stimulus A when presented on NA trials. A *post hoc* simulation of a reinforcement-based configural model supported our assertion.

## INTRODUCTION

Discrimination learning theory has been continuously challenged by the ambiguous-cue problem. This deceptively simple problem involves three stimuli arranged in two simultaneous discriminations. The critical feature is that the reinforced stimulus (S+) in one discrimination serves as the non-reinforced stimulus (S-) in the other, hence the ambiguous-cue problem (Thompson, 1954). The three stimuli are: P, the positive or always reinforced stimulus; N, the negative or always non-reinforced stimulus; and A, the ambiguous stimulus, which is negative or positive depending on whether it is presented together with P or N, respectively. The simultaneous discriminations are usually denoted as PA trials and NA trials.

Despite some early controversy about which discrimination (PA vs. NA) is easier to learn (cf. Thompson, 1954; Leary, 1958; Zeaman and House, 1962; Fletcher et al., 1968; Boyer and Polidora, 1972; Fletcher and Garske, 1972), currently it is uncontroversial that, if salient stimuli are used as cues, performance on PA trials is less accurate than on NA trials—the ambiguous-cue effect. This pattern has been observed in a variety of species, including pigeons, monkeys, honeybees, children, and adults with mental retardation (e.g., Fletcher and Garske, 1972; Richards and Marcattilio, 1975; Hall, 1980; Couvillon and Bitterman, 1986; Urcuioli and Michalek, 2007; Nardi, 2009).

Two main accounts have been proposed to explain the effect, both stressing the status of A as an excitatory stimulus in both discriminations. The dominant and initial approach, known as the interfering cue hypothesis (e.g., Zeaman and House, 1962; Fletcher and Garske, 1972) proposes that whereas PA trials involve an approach-approach conflict (P is always reinforced and A is partially reinforced across discriminations), no conflict is evident on NA trials (A is partially reinforced and N is never reinforced). In other words, PA trials involve a choice between stimuli with differential but positive value and NA trials involve a choice between a stimulus with positive value and a stimulus with either negative or zero value, thus making the NA discrimination much easier.

More recently, Urcuioli and Michalek (2007) proposed that value transfer may also contribute to the effect. Value transfer theory (von Fersen et al., 1991) proposes that associative value transfers from one stimulus to the other in a simultaneous discrimination and subsequent research has shown that value transfer (a) does indeed occur (Zentall and Sherburne, 1994), and (b) it occurs only from the S+ to the S- and not in the opposite direction (Clement et al., 1998; for a possible role of the hippocampus in value transfer, see Van Elzakker et al., 2003). One possible mechanism for this “transfer” is second-order conditioning (Davis, 1992). Assuming that in a simultaneous discrimination the animals observe the stimuli sequentially (Wright and Sands, 1981), then the S- is functionally followed by the S+ on approximately one half of the trials. In Pavlovian language, CS2 is followed by CS1 and then by the US. Such second-order conditioning should impart “value” to CS2 (Zentall et al., 1996; Zentall, 2004). According to this theory, the value of any stimulus in a simultaneous discrimination is the sum of its direct value (conveyed by reinforcement) plus the indirect value it receives through positive value transfer (for what appears to be an anticipation of value transfer theory, see Leary, 1956, 1958).

To test whether or not the value transfer mechanism plays a role in an ambiguous-cue task, Urcuioli and Michalek (2007, Experiment 1) devised a test involving partially reinforcing P on PA trials. With this schedule, both P and A would be reinforced 50% of the time, but A would also acquire some value from P via value transfer. Accordingly, if value transfer operates, the overall value of A would exceed that of P, leading to the peculiar prediction of below chance performance on PA trials (i.e., animals should select the S- more frequently than the S+ on PA trials). In contrast, the interfering cue hypothesis which considers only direct values, predicts that if P is reinforced 50% of the times and A is also reinforced 50% of the times (although admittedly across trials), subjects should be indifferent between P and A. On NA trials, both accounts predict high levels of accuracy. Urcuioli and Michalek’s (2007) pigeons attained accuracies of 9.2% on partially reinforced PA trials and 99.5% on NA trials, thus suggesting that value transfer contributes to ambiguous-cue performance and seriously questioning the predictions of the interfering cue hypothesis.

Given that the results of Urcuioli and Michalek (2007) stand alone in the ambiguous-cue literature in questioning the dominant account and simultaneously propose an alternative mechanism, this study re-examined the controversy using a new model species, the European starling (*Sturnus vulgaris*).

## EXPERIMENT 1

**FIGURE 1:**
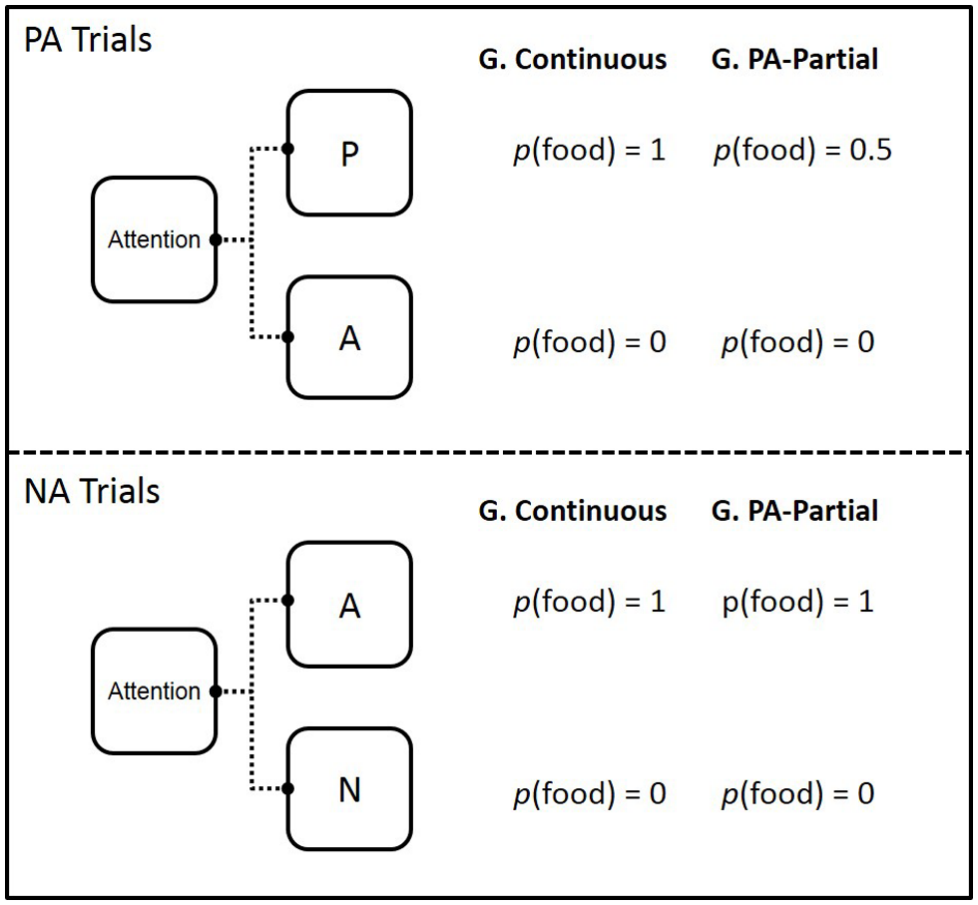
**Design of Experiments 1 and 2.** The colors (red, green, and blue) associated with stimuli P, A, and N were counterbalanced across subjects. P, A, and N appeared equally often on the left and right keys; G denotes Group and *p* denotes probability.

This experiment was a systematic replication of Groups Continuous and PA-Partial ran by Urcuioli and Michalek (2007, Experiment 1). The main changes were the number of trials scheduled per daily session (60 in Urcuioli and Michalek, 2007, 360 here) and the model species (pigeons and starlings, respectively). A schematic of the design of this experiment is presented in **Figure 1**.

### MATERIALS AND METHODS

#### Subjects

Twelve experimentally naïve, wild-caught adult European starlings (*S. vulgaris*) participated in the experiment (English Nature license No. 20010082). During the experiment, starlings were housed in pairs in indoor cages which were visually, but not acoustically isolated. Each room contained two cages that served both as home and experimental cages. Indoor temperatures ranged from 15 to 18°C and lights followed a 12:12 dark schedule with light from 07h00 to 19h00, and gradual transitions at dawn and dusk. After each daily experimental session, starlings had 4 h (13h00–17h00) of free access to turkey crumbs, Orlux© Remiline universal granules and 10 mealworms daily, and social interaction with the respective cage-partner. This regime maintains starlings at approximately 90% of their free-feeding weight (Bateson, 1993). All procedures were in accordance with the University of Oxford’s animal care guidelines.

When not participating in an experiment, the starlings were housed together in two outdoor aviaries. While in the aviary, they received ad libitum food, a mixture of turkey crumbs, Orlux pellets and mealworms (*Tenebrio sp*.). Drinking and bathing water was always available and replaced daily. After the experiment, starlings were returned to the communal aviary. All subjects were released into the wild after participating in three similar experiments, and following at least 2 weeks of re-acclimatization to natural light in the outdoor aviary.

#### Apparatus

**FIGURE 2:**
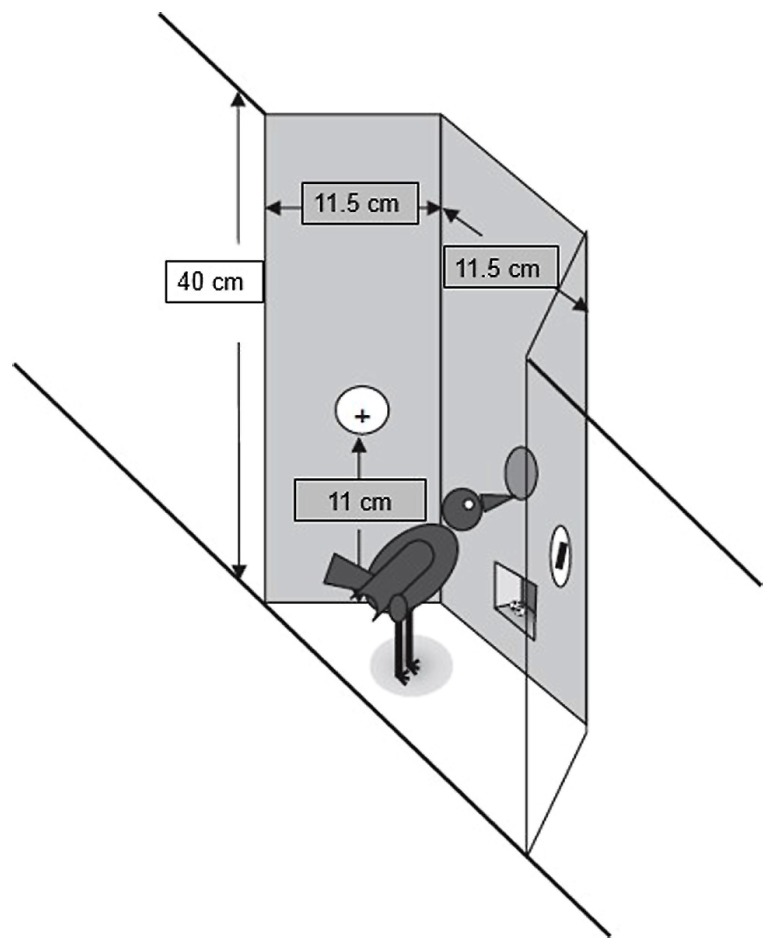
**Schematic representation of the experimental panel used in the experiments.** Adapted with permission from Shapiro et al. (2008).

Each indoor cage [135 × 78.4 × 80 cm (l × w × h)] was composed of two units, vertically mounted (80 cm each). Each unit included two experimental areas separated by a common middle section. Each experimental area had a panel attached 10 cm above the floor. The panel was 40 cm tall with three sections: a middle sub panel, facing the cage (11.5 cm wide), and two side subpanels (same length) attached to the cage at a 120°angle from the center subpanel. The middle subpanel had one response key in the center (11 cm from the bottom), and the food hopper (2.5 cm from the bottom), that was connected to the pellet dispenser (Campden Instruments^®^) containing 20 mg BioServ^®^ precision pellets. Each side subpanel had one response key in the center (11 cm from the bottom). Every key was composed by a 16 LED matrix that could display 16 different symbols in seven possible colors. A schematic of the panel is presented in **Figure 2**. A computer in an adjacent room controlled all experimental events.

#### Procedure

Prior to the start of the experiment, starlings were randomly divided into two groups: Group Continuous and Group PA-Partial.

***Preliminary training.*** All starlings received autoshaping training sessions to ensure that they pecked all keys and hues used in this experiment. Autoshaping particulars were as described in Aw et al. (2011, Appendix 1). At the end of this phase, all starlings reliably pecked the white center key, and the red, green, and blue side-keys (all 4 × 4 LEDs) for reinforcement.

***Discrimination training.*** Next, all starlings were trained on an ambiguous-cue discrimination, in which red, green, and blue served as discriminative stimuli. All trials began with the white center key flashing (700 ms ON, 300 ms OFF). A single peck to this attention key switched its light off and immediately produced two discriminative stimuli on the side keys. A single peck to either key turned both stimuli off and produced either two food pellets or advanced the program to the next trial, depending on whether the response was correct or incorrect, respectively. Consecutive trials were always separated by a 40 s inter-trial interval (ITI) during which no operant panel lights were illuminated.

The P and A stimuli were presented together on half of the trials (PA trials) and the N and A stimuli (NA trials) were presented together on the remaining half. On PA trials, choosing P was always reinforced for starlings in Group Continuous but reinforced only half of the time for starlings in Group PA-Partial, whereas choice of A was always unreinforced in both groups. On NA trials, choosing A was always reinforced and choosing N was always unreinforced for both groups.

Starlings received one session per day for 18 days. Sessions ended after 360 trials or 5.5 h from the session start (7:30 am), whichever came first. Each completed session consisted of 180 PA trials and 180 NA trials, all with side key allocation counterbalanced across trials. PA and NA trials occurred in a pseudorandom order with the constraint that no more than three of each trial type could occur consecutively. The hues (red, green, and blue) used for the stimuli P, A, and N, were counterbalanced within each group.

#### Data analysis

Prior to analysis, all proportion data were normalized using an arcsine square root transformation, (Grafen and Hails, 2002). A Type-1 error rate of 0.05 was adopted for all statistical comparisons. Of the 12 starlings, one bird from Group Continuous was dropped from the study due to an injury during the initial sessions of discrimination training.

### RESULTS AND DISCUSSION

**FIGURE 3:**
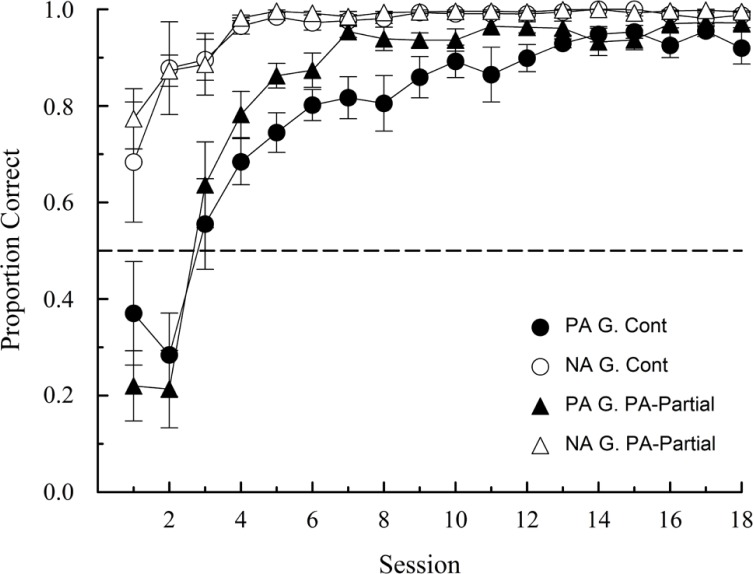
**Proportion of correct choices (±1 SEM) on PA and NA trials during acquisition in Experiment 1.** G denotes Group.

**Figure 3** shows the mean proportion of correct responses on PA and NA trials for both groups. As predicted, both groups revealed faster acquisition on NA than on PA trials. However, the pattern of results for PA trials in both groups is noticeably different from that reported by Urcuioli and Michalek (2007), with both groups reaching high levels of accuracy.

A mixed analysis of variance (ANOVA) with group, trial type and session as factors revealed a significant main effect of group [*F*(1,9) = 6.72, *p* = 0.029, $\eta_{p}^{2}$ = 0.43], trial type [*F*(1,9) = 54.87, *p* < 0.001, $\eta_{p}^{2}$ = 0.86], and session [*F*(17,153) = 76.40, *p* < 0.001, $\eta_{p}^{2}$ = 0.90], and a significant interaction between trial type and session [*F*(17,153) = 8.56, *p* < 0.001, $\eta_{p}^{2}$ = 0.49], the latter confirming a PA-NA difference in the rate of acquisition as suggested by a visual inspection of **Figure 3**. All other interactions were non-significant.

Subsequent analyses over the last four sessions, when performances appeared to have stabilized, revealed that an ambiguous-cue effect was still present only in Group PA-Partial. The average proportions of correct choices in Group Continuous were 0.94 (SEM: 0.016) and 0.99 (SEM: 0.005) on PA and NA trials, respectively. The corresponding choice proportions for Group PA-Partial were 0.96 (SEM: 0.008) and 0.99 (SEM: 0.002). These differences were non-significant in Group Continuous [*t*(4) = -2.25, *p* = 0.088] and statistically significant in Group PA-Partial [*t*(5) = -8.29, *p* < 0.001, *d* = -3.43]. Importantly, between-group comparisons over the last four sessions returned no statistically significant differences either on PA [*t*(9) = -0.94, *p* = 0.374] or NA trials [*t*(9) = -0.81, *p* = 0.440].

To summarize, the results observed on NA trials are consistent with what has been repeatedly reported in the literature (e.g., Fletcher et al., 1968; Richards and Marcattilio, 1975; Hall, 1980; Urcuioli and Michalek, 2007). On the contrary, starlings’ accuracy on PA trials reached levels that, to our knowledge, have never been reported in a standard ambiguous-cue discrimination task, at least in birds. Richards and Marcattilio (1975) and Urcuioli and Michalek (2007) did find high levels of accuracy on PA trials but only when NA trials were partially reinforced.

In fact, the terminal performance of both groups conflicts with the predictions of the interfering cue hypothesis and value transfer theory. On the one hand, the interfering cue hypothesis predicts a much larger PA-NA difference in both groups. In particular, NA performance should be high in both groups (as we indeed observed) and PA performance should be above chance (but below NA performance) in Group Continuous and at chance in Group PA-Partial. On the other hand, value transfer theory predicts high accuracy levels on NA trials in both groups, but chance and below chance performance on PA trials in Groups Continuous and PA-Partial, respectively. We observed none of these patterns. Nonetheless, the pattern of acquisition observed in the first few sessions (lower PA than NA performance) is predicted by both accounts, but not fully consistent with either of them (e.g., PA performance in Group Continuous).

A noticeable procedural difference between our study and the vast majority of studies reported in the literature is the number of trials per session. For instance, Urcuioli and Michalek (2007) included 60 trials per session compared to the 360 trials used here. At the end of session 4 (with a maximum of 720 trials per discrimination completed), the starling’s PA performance was already significantly above chance in both groups. At roughly the same number of training trials, Urcuioli and Michalek’s (2007) pigeons were either at chance (46.25% correct, Group Continuous) or below chance (9.17% correct, Group PA-Partial).

Naturally, we cannot guarantee that the massed daily presentation of 180 PA and NA trials is functionally the same as presenting 180 trials over six consecutive days. In fact, it is certainly not equivalent in terms of the dynamics of learning and memory (e.g., Roberts, 1974; Adams, 1982; Yin et al., 1994).

Experiment 2 attempts to eliminate these possible confounds in the comparison between studies by closely following the procedure used by Urcuioli and Michalek (2007).

## EXPERIMENT 2

To have a clearer idea of how starlings’ performance in the ambiguous-cue problem compare to other species and whether or not their performance questions the major explanatory accounts of the effect, we ran a second experiment comprising only 60 trials per daily session.

### MATERIALS AND METHODS

Twelve experimentally naïve, wild-caught adult starlings participated in this experiment. Their licensing, housing and training conditions were as described in Experiment 1, except that 36 daily sessions were run, each composed of 60 trials.

### RESULTS AND DISCUSSION

**FIGURE 4:**
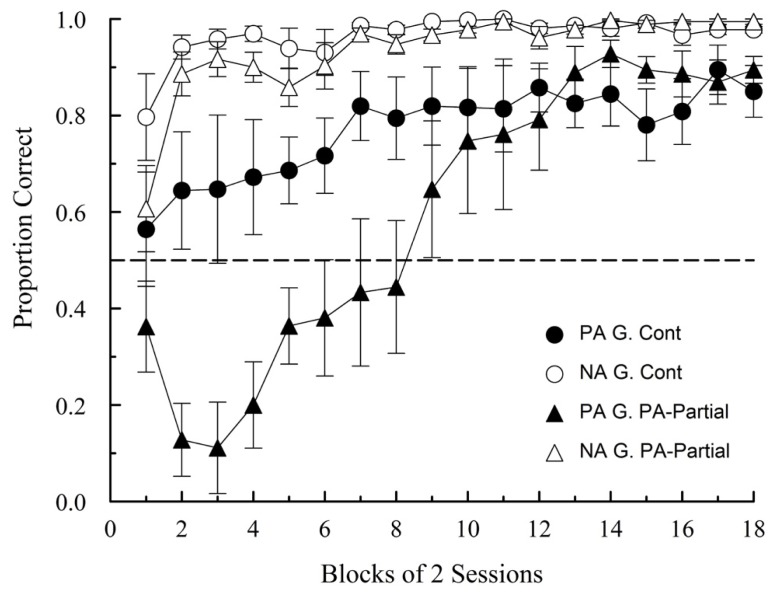
**Proportion of correct choices (±1 SEM) on PA and NA trials during acquisition in Experiment 2.** G denotes Group.

The overall pattern of results observed in this experiment was similar to that obtained in Experiment 1. Both groups reached relatively high levels of accuracy both in PA and NA trials, albeit at different stages of training. The mean proportion of correct responses on PA and NA trials for both groups over blocks of two sessions is shown in **Figure 4**.

A mixed ANOVA with Group, Trial type and Block as factors revealed a marginally significant main effect of group [*F*(1,10) = 4.78, *p* = 0.054, $\eta_{p}^{2}$ = 0.32], and significant main effects of trial type [*F*(1,10) = 39.37, *p* < 0.001, $\eta_{p}^{2}$ = 0.80], and block [*F*(17,170) = 19.31, *p* < 0.001, $\eta_{p}^{2}$ = 0.66]. The interactions Group × Block, Trial Type × Block, and Group × Trial Type × Block were also statistically significant [*F*(17,170) = 5.99, *p* < 0.001, $\eta_{p}^{2}$ = 0.37; *F*(17,170) = 4.67, *p* < 0.001, $\eta_{p}^{2}$ = 0.32; and *F*(17,170) = 2.55, *p* = 0.001, $\eta_{p}^{2}$ = 0.20, respectively]. This pattern of results confirms once again a PA-NA difference in the rate of acquisition, but also what appears to be a strong effect of partial reinforcement on PA trials. A retardation of acquisition is clear in Group PA-Partial relative to Group Continuous on PA trials. The drop below chance in the first few sessions could be potentially caused by a value transfer mechanism, but asymptotic performance suggests that such mechanism, if it in fact operated early on, was subsequently overcome by other factors. A mixed ANOVA with Group and Block as factors restricted to PA performance confirmed a significant Group × Block interaction [*F*(17,170) = 5.02, *p* < 0.001, $\eta_{p}^{2}$ = 0.33], as well a significant main effect of Block [*F*(17,170) = 12.12, *p* < 0.001, $\eta_{p}^{2}$ine-formula> = 0.55]. The effect of Group was non-significant [*F*(1,10) = 3.45, *p* = 0.093].

Subsequent analyses over the last four sessions, revealed that, as in Experiment 1, a significant ambiguous-cue effect was present only on Group PA-Partial. [Group Continuous: *t*(5) = -1.914, *p* = 0.114; Group PA-Partial: *t*(5) = -4.40, *p* = 0.007, *d* = -1.86]. The average proportions of correct choices in Group Continuous were 0.87 (SEM: 0.051) and 0.98 (SEM: 0.007) on PA and NA trials, respectively. The corresponding proportions for Group PA-Partial were 0.88 (SEM: 0.035) and 0.99 (SEM: 0.006). Between-group comparisons over the same sessions revealed no statistically significant differences on PA trials [*t*(10) = 0.02, *p* = 0.981], but a significant accuracy difference on NA trials [*t*(10) = -2.26, *p* = 0.047, *d* = -1.43], which is numerically quite small as a quick scan of **Figure 4** shows (1.65%, on average, to be exact). One evident difference between experiments is that PA accuracy was generally lower in this experiment (cf. **Figures 3 and 4**), which is most probably due to the large difference in the amount of training.

Overall, this experiment confirmed the general findings of Experiment 1, although it also revealed a clear effect of partial reinforcement on PA trials that was probably masked in Experiment 1 by the larger number of daily trials. In fact, this was the only hint that a value transfer mechanism could potentially be operating early on. Nonetheless, starlings’ asymptotic accuracy on PA trials defies both value transfer theory and the interfering cue hypothesis as complete explanations of the ambiguous-cue problem. While a significant PA-NA difference is still observed in Group PA-Partial, the accuracy levels attained by both groups cannot be explained by either theoretical account.

## GENERAL DISCUSSION

The present findings are hard to reconcile with the major theoretical accounts of the ambiguous-cue effect, namely the interfering cue hypothesis and value transfer theory. The terminal PA–NA differences observed in both experiments are unanticipated by either account, thus suggesting that either starlings are better able to overcome the typical difficulties posed by the task or they are less susceptible to them. Although an interpretation based solely on the interfering cue hypothesis or value transfer might be insufficient to explain this result, starlings did show slower acquisition on PA trials and almost perfect performance on NA trials, which seems to indicate that some of the mechanisms proposed by such accounts may indeed operate early on.

Particularly noticeable is the difference between our results and those reported by Urcuioli and Michalek (2007, Experiment 1). Whereas their pigeons in Groups Continuous and PA-Partial showed chance and below chance performance on PA trials, respectively, our starlings learned the PA discrimination to high levels of accuracy in both groups. Of course, the use of different species (pigeons vs. starlings) makes comparisons between experiments complicated and we cannot exclude that that may indeed be one of the reasons for such discrepancies. Unfortunately, comparisons between species have been hampered by differences in research programs in comparative psychology and behavioral biology (Shettleworth, 1993), but the scenario is changing rapidly (Shettleworth, 2009, 2012). Regarding the comparison of the cognitive and learning abilities of pigeons and starlings very little is known, although both species have been exposed to similar tasks by different research groups, mainly in the domains of timing (e.g., Gibbon, 1977; Gibbon et al., 1988; Brunner et al., 1992; Kacelnik and Brunner, 2002), and decision making (e.g., Schuck-Paim and Kacelnik, 2002; Shapiro et al., 2008; Lagorio and Hackenberg, 2010; Mazur, 2010; Vasconcelos et al., 2010; Kacelnik et al., 2011; Aw et al., 2012).

Another important difference between studies is related to the operant panels used. At first sight, our angled panels (cf. **Figure 2**) may have precluded value transfer from occurring. If, as proposed, the mechanism supporting value transfer is second-order conditioning (CS2 followed by CS1 and then by the US; cf. Zentall et al., 1996; Zentall, 2004), this is much more likely to occur with flat panels (as those used by Urcuioli and Michalek, 2007) which facilitate sequential observation of stimuli than with angled panels where the stimuli are more likely to be observed simultaneously provided, as we did, the birds are centrally located (for a similar discussion on the effect of apparatus variations on behavioral data, see Katz et al., 2008). Be that as it may, we did observe below chance performance on partially reinforced PA trials in the first few sessions of Experiment 2, which may render the argument moot.

It is interesting to note that transitive inference tasks with non-human animals are basically a set of ambiguous-cue problems. A typical task involves four intermixed, and partially overlapping simultaneous discriminations: A+ B-, B+ C-, C+ D-, and D+ E-, and animals are usually able to learn them (e.g., McGonigle and Chalmers, 1977; Gillan, 1981; D’Amato and Colombo, 1990; von Fersen et al., 1991; Mcgonigle and Chalmers, 1992; Boysen et al., 1993; Higa and Staddon, 1993; Rapp et al., 1996; Siemann et al., 1996; Treichler and Van Tilburg, 1996; Dusek and Eichenbaum, 1997; Wynne, 1997; Van Elzakker et al., 2003; Buckmaster et al., 2004; Lazareva et al., 2004; Lazareva and Wasserman, 2006). Accuracy levels are generally high in all discriminations, but follow a U-shaped function, known as the *serial position effect* (Bryant and Trabasso, 1971; Woocher et al., 1978) with the discriminations at each end (A+ B- and D+ E-) better solved than the other ones.

Curiously, the most successful transitive inference models assume that each stimulus is, to some extent, bound to the context in which they occur (see, Rescorla, 1972; Whitlow and Wagner, 1972; Rescorla, 1973). In particular, Wynne’s configural model (Wynne, 1995, 1998) and Siemann–Delius model (Delius and Siemann, 1998; see also Siemann and Delius, 1998) propose that a stimulus is not functionally the same when presented in different discriminations: it has an elemental value but also a configural value dependent on other stimuli simultaneously present.

Returning to the ambiguous-cue task, this reasoning means that stimulus A is not functionally the same on PA and NA trials because of different configural cues (P and N, respectively). If this is indeed the case, then accuracy on PA trials should increase as animals learn the informational value of such cues. Given that this argument is consistent with our results, we fitted Wynne’s configural model to the results obtained in Experiment 2. This model is well known in the literature and we will not detail it here (for mathematical details, see Wynne, 1995, 1998; Lazareva et al., 2004; Vasconcelos, 2008). The data of each starling were fitted individually, using the full sequence of trials presented during training. Because the model is not intended to account for acquisition data we searched for the combination of parameters that provides the best-fitting performance to the asymptotic performance (last four sessions) by minimizing the root-mean-square error (RMSE).

**FIGURE 5:**
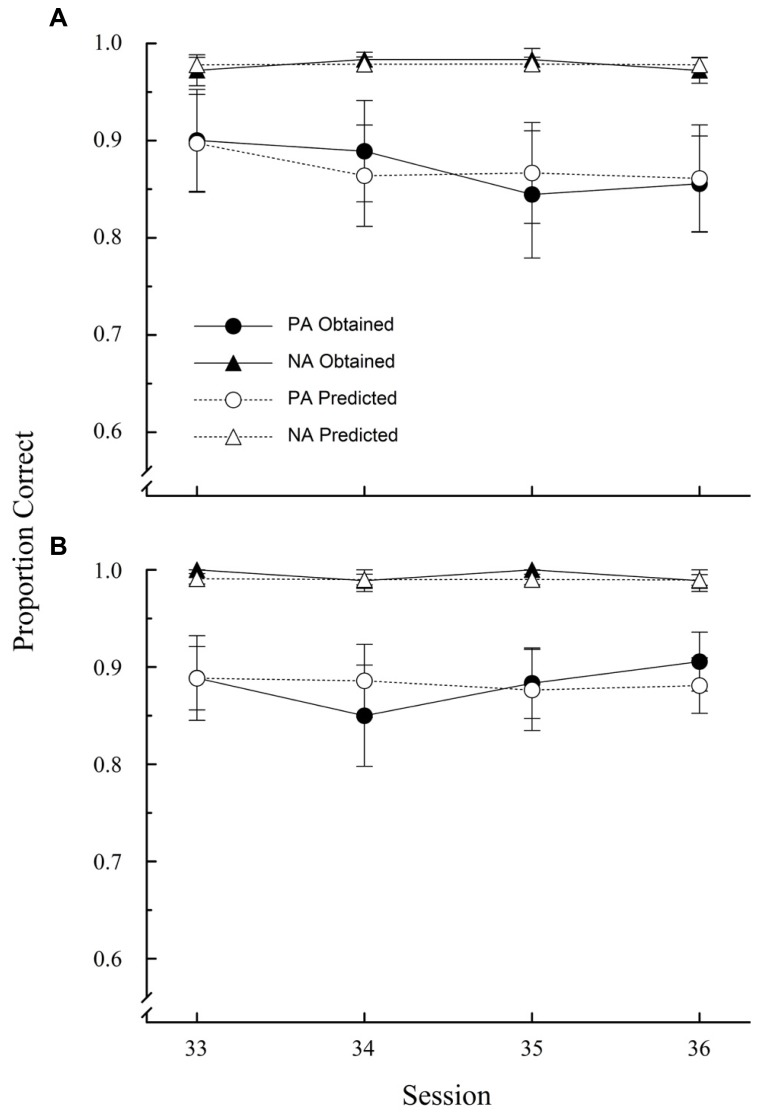
**Starlings’ asymptotic accuracy over the last four sessions (black symbols) and simulated accuracy (white symbols) according to Wynne’s configural model ( ± 1 SEM); (A) Group Continuous; (B) Group PA-Partial**.

**Figures 5A,B** depict the results of these simulations for Groups Continuous and PA-Partial, respectively. Overall, the predictions closely matched asymptotic performance, with average RMSEs of 0.03 and 0.04. This suggests that attention to configural cues may indeed be relevant to solve the ambiguous-cue problem. What remains to be answered is why Urcuioli and Michalek’s (2007) pigeons seemed to ignore such cues, despite the ensuing decrease in food intake. One possibility rests again on the panels used. Perhaps configural cues are harder to learn with flat panels, where simultaneous observation of both stimuli is less likely. Still, without further tests, this belongs to the realm of speculation. An established fact, however, is that under some circumstances pigeons are able to attend to the gestalt of whole stimulus displays (i.e., configural learning; e.g., Wright, 1997; Katz et al., 2008) and to learn the more complex transitive inference task despite a rather slow acquisition (for a review, see Vasconcelos, 2008).

For the moment, it is clear that European starlings’ terminal performance on the ambiguous-cue problem challenge the major theoretical accounts of the ambiguous-cue effect. Neither the interfering cue hypothesis nor value transfer theory can predict high PA accuracy, particularly in Group PA-Partial. Yet, the initial acquisition pattern observed in both experiments is at least partially consistent with such accounts. Perhaps the inconsistencies between this and previous studies are due to species differences (to our knowledge this was the first time starlings were tested on this task), differences in apparatus, or a combination of both.

Whilst the reasons for the discrepancies between starlings’ and pigeons’ performance await for further tests, this article will have served its function if the reader is at least partially convinced that current accounts of the ambiguous-cue problem are incomplete and that the dynamics of acquisition is not yet fully understood. Hopefully, this will lead to new theorizing about the problem, standardized procedures and informative new experiments.

## AUTHOR CONTRIBUTIONS

Marco Vasconcelos and Tiago Monteiro jointly designed the experiments and collected the data, Marco Vasconcelos processed and analyzed the data, Marco Vasconcelos and Tiago Monteiro jointly wrote the manuscript.

## Conflict of Interest Statement

The authors declare that the research was conducted in the absence of any commercial or financial relationships that could be construed as a potential conflict of interest.

## References

[B1] Adams C. D. (1982). Variations in the sensitivity of instrumental responding to reinforcer devaluation. *Q. J. Exp. Psychol. B* 34 77–98 10.1080/14640748208400878

[B2] Aw J. Monteiro T. Vasconcelos M. Kacelnik A. (2012). Cognitive mechanisms of risky choice: is there an evaluation cost? *Behav. Processes* 89 95–103 10.1016/j.beproc.2011.09.007 22001371

[B3] Aw J. M. Vasconcelos M. Kacelnik A. (2011). How costs affect preferences: experiments on state dependence, hedonic state and within-trial contrast in starlings. *Anim. Behav.* 81 1117–1128 10.1016/j.anbehav.2011.02.015

[B4] Bateson M. (1993). *Currencies for Decision Making: the Foraging Starling as a Model Animal*. Ph.D. thesis, University of Oxford, Oxford

[B5] Boyer W. N. Polidora V. J. (1972). An analysis of the solution of PAN ambiguous-cue problems by rhesus monkeys. *Learn. Motiv.* 3 325–333 10.1016/0023-9690(72)90028-8

[B6] Boysen S. T. Berntson G. G. Shreyer T. A. Quigley K. S. (1993). Processing of ordinality and transitivity by chimpanzees (*Pan troglodytes*). *J. Comp. Psychol.* 107 208–215 10.1037/0735-7036.107.2.208 8370275

[B7] Brunner D. Kacelnik A. Gibbon J. (1992). Optimal foraging and timing processes in the starling, *Sturnus vulgaris*: effect of inter-capture interval. *Anim. Behav.* 44 597–613 10.1016/S0003-3472(05)80289-1

[B8] Bryant P. E. Trabasso T. (1971). Transitive inferences and memory in young children. *Nature* 232 456–458 10.1038/232456a0 4937205

[B9] Buckmaster C. A. Eichenbaum H. Amaral D. G. Suzuki W. A. Rapp P. R. (2004). Entorhinal cortex lesions disrupt the relational organization of memory in monkeys. *J. Neurosci.* 24 9811–9825 10.1523/JNEUROSCI.1532-04.2004 15525766 PMC6730224

[B10] Clement T. S. Weaver J. E. Sherburne L. M. Zentall T. R. (1998). Simultaneous discrimination learning in pigeons: value of S- affects the relative value of its associated S+. *Q. J. Exp. Psychol. B* 51 363–378 10.1080/713932684 9854439

[B11] Couvillon P. A. Bitterman M. E. (1986). Performance of honeybees in reversal and ambiguous-cue problems: tests of a choice model. *Anim. Learn. Behav.* 14 225–231 10.3758/BF03200062

[B12] D’Amato M. R. Colombo M. (1990). The symbolic distance effect in monkeys (*Cebus apella*). *Anim. Learn. Behav.* 18 133–140 10.3758/BF03205250

[B13] Davis H. (1992). “Logical transitivity in animals,” in *Cognitive Aspects of Stimulus Control* eds Honig W. K. Fetterman J. G. (Hillsdale, NJ: Erlbaum) 405–429

[B14] Delius J. D. Siemann M. (1998). Transitive responding in animals and humans: exaptation rather than adaptation? *Behav. Processes* 42 107–137 10.1016/S0376-6357(97)00072-7 24897458

[B15] Dusek J. A. Eichenbaum H. (1997). The hippocampus and memory for orderly stimulus relations. *Proc. Natl. Acad. Sci. U.S.A.* 94 7109–7114 10.1073/pnas.94.13.7109 9192700 PMC21293

[B16] Fletcher H. J. Garske J. P. (1972). Response competition in monkeys’ solution of PAN ambiguous-cue problems. *Learn. Motiv.* 3 334–340 10.1016/0023-9690(72)90029-X

[B17] Fletcher H. J. Grogg T. M. Garske J. P. (1968). Ambiguous-cue problem performance of children, retardates, and monkeys. *J. Comp. Physiol. Psychol.* 66 477–482 10.1037/h0026343 4972575

[B18] Gibbon J. (1977). Scalar expectancy theory and Weber’s law in animal timing. *Psychol. Rev.* 84 279–325 10.1037/0033-295X.84.3.279

[B19] Gibbon J. Church R. M. Fairhurst S. Kacelnik A. (1988). Scalar expectancy theory and choice between delayed rewards. *Psychol. Rev.* 95 102–114 10.1037/0033-295X.95.1.102 3353474

[B20] Gillan D. J. (1981). Reasoning in the chimpanzee: II. transitive inference. *J. Exp. Psychol. Anim. Behav. Process.* 7 150–164 10.1037/0097-7403.7.2.150

[B21] Grafen A. Hails R. (2002). *Modern Statistics for the Life Sciences*. New York: Oxford University Press

[B22] Hall G. (1980). An investigation of ambiguous-cue learning in pigeons. *Anim. Learn. Behav.* 8 282–286 10.3758/BF03199607

[B23] Higa J. J. Staddon J. E. R. (1993). Transitive inference in multiple conditional discriminations. *J. Exp. Anal. Behav.* 59 265–291 10.1901/jeab.1993.59-265 8454956 PMC1322042

[B24] Kacelnik A. Brunner D. (2002). Timing and foraging: gibbon’s scalar expectancy theory and optimal patch exploitation. *Learn. Motiv.* 33 177–195 10.1006/lmot.2001.1110

[B25] Kacelnik A. Vasconcelos M. Monteiro T. Aw J. (2011). Darwin’s “tug-of-war” vs. starlings’ “horse-racing”: how adaptations for sequential encounters drive simultaneous choice. *Behav. Ecol. Sociobiol.* 65 547–558 10.1007/s00265-010-1101-2

[B26] Katz J. S. Bodily K. D. Wright A. A. (2008). Learning strategies in matching to sample: If-then and configural learning by pigeons. *Behav. Processes* 77 223–230 10.1016/j.beproc.2007.10.011 18079071 PMC2290969

[B27] Lagorio C. H. Hackenberg T. D. (2010). Risky choice in pigeons and humans: a cross-species comparison. *J. Exp. Anal. Behav.* 93 27–44 10.1901/jeab.2010.93-27 20676266 PMC2801539

[B28] Lazareva O. F. Smirnova A. A. Bagozkaja M. S. Zorina Z. A. Rayevsky V. V. Wasserman E. A. (2004). Transitive responding in hooded crows requires linearly ordered stimuli. *J. Exp. Anal. Behav.* 82 1–19 10.1901/jeab.2004.82-1 15484868 PMC1284988

[B29] Lazareva O. F. Wasserman E. A. (2006). Effect of stimulus orderability and reinforcement history on transitive responding in pigeons. *Behav. Processes* 72 161–172 10.1016/j.beproc.2006.01.008 16460886

[B30] Leary R. W. (1956). The rewarded, the unrewarded, the chosen, and the unchosen. *Psychol. Rep.* 2 91–97 10.2466/PR0.2.3.91-97

[B31] Leary R. W. (1958). The learning of ambiguous cue-problems by monkeys. *Am. J. Psychol.* 71 718–724 10.2307/1420329 13627280

[B32] Mazur J. E. (2010). Distributed versus exclusive preference in discrete-trial choice. *J. Exp. Psychol. Anim. Behav. Process.* 36 321–333 10.1037/a0017588 20658863 PMC2911998

[B33] Mcgonigle B. Chalmers M. (1992). Monkeys are rational! *Q. J. Exp. Psychol. B* 45 189–228 10.1080/14640749208401017

[B34] McGonigle B. O. Chalmers M. (1977). Are monkeys logical? *Nature* 267 694–696 10.1038/267694a0 406574

[B35] Nardi N. M. (2009). *An Investigation Into the Ambiguous Cue Problem with Pigeons*. Master thesis, California State University, Chico

[B36] Rapp P. R. Kansky M. T. Eichenbaum H. (1996). Learning and memory for hierarchical relationships in the monkey: effects of aging. *Behav. Neurosci.* 110 887–897 10.1037/0735-7044.110.5.887 8918992

[B37] Rescorla R. A. (1972). “Configural” conditioning in discrete-trial bar pressing. *J. Comp. Physiol. Psychol.* 79 307–317 10.1037/h0032553 5025999

[B38] Rescorla R. A. (1973). Evidence for “unique stimulus” account of configural conditioning. *J. Comp. Physiol. Psychol.* 85 331–338 10.1037/h0035046

[B39] Richards R. W. Marcattilio A. J. (1975). Intermittency of reinforcement during NA trials and performance on the ambiguous-cue problem. *Can. J. Psychol.* 29 210–223 10.1037/h0082027

[B40] Roberts W. A. (1974). Spaced repetition facilitates short-term retention in the rat. *J. Comp. Physiol. Psychol.* 86 164–171 10.1037/h0035965

[B41] Schuck-Paim C. Kacelnik A. (2002). Rationality in risk-sensitive foraging choices by starlings. *Anim. Behav.* 64 869–879 10.1006/anbe.2002.2003

[B42] Shapiro M. S. Siller S. Kacelnik A. (2008). Simultaneous and sequential choice as a function of reward delay and magnitude: normative, descriptive and process-based models tested in the European Starling (*Sturnus vulgaris*). *J. Exp. Psychol. Anim. Behav. Process.* 34 75–93 10.1037/0097-7403.34.1.75 18248116

[B43] Shettleworth S. J. (1993). Where is the comparison in comparative cognition? Alternative research programs. *Psychol. Sci.* 4 179–184 10.1111/j.1467-9280.1993.tb00484.x

[B44] Shettleworth S. J. (2009). The evolution of comparative cognition: is the snark still a boojum? *Behav. Processes* 80 210–217 10.1016/j.beproc.2008.09.001 18824222

[B45] Shettleworth S. J. (2012). “Darwin, Tinbergen, and the evolution of comparative cognition,” in *Oxford Handbook of Comparative Evolutionary Psychology* eds Vonk J. Shackelford T. K. (New York, NY: Oxford University Press) 529–546

[B46] Siemann M. Delius J. D. (1998). Algebraic learning and neural network models for transitive and non-transitive responding. *Eur. J. Cogn. Psychol.* 10 307–334 10.1080/713752279

[B47] Siemann M. Delius J. D. Wright A. A. (1996). Transitive responding in pigeons: influences of stimulus frequency and reinforcement history. *Behav. Processes* 37 185–195 10.1016/0376-6357(96)000204 24897441

[B48] Thompson R. (1954). Approach versus avoidance in an ambiguous-cue discrimination problem in chimpanzees. *J. Comp. Physiol. Psychol.* 47 133–135 10.1037/h0060851 13152227

[B49] Treichler F. R. Van Tilburg D. (1996). Concurrent conditional discrimination tests of transitive inference by macaque monkeys: list linking. *J. Exp. Psychol. Anim. Behav. Process.* 22 105–117 10.1037/0097-7403.22.1.105 8568492

[B50] Urcuioli P. J. Michalek S. (2007). Value transfer contributes to ambiguous-cue discrimination learning. *Psychon. Bull. Rev.* 14 658–662 10.3758/BF03196817 17972729

[B51] Van Elzakker M. O’reilly R. C. Rudy J. W. (2003). Transitivity, flexibility, conjunctive representations, and the hippocampus. I. An empirical analysis. *Hippocampus* 13 334–340 10.1002/hipo.10083 12722974

[B52] Vasconcelos M. (2008). Transitive inference in non-human animals: an empirical and theoretical analysis. *Behav. Processes* 78 313–334 10.1016/j.beproc.2008.02.017 18423898

[B53] Vasconcelos M. Monteiro T. Aw J. Kacelnik A. (2010). Choice in multi-alternative environments: a trial-by-trial implementation of the sequential choice model. *Behav. Processes* 84 435–439 10.1016/j.beproc.2009.11.010 19948210

[B54] von Fersen L. Wynne C. D. L. Delius J. D. Staddon J. E. R. (1991). Transitive inference formation in pigeons. *J. Exp. Psychol. Anim. Behav. Process.* 17 334–341 10.1037/0097-7403.17.3.334

[B55] Whitlow J. W. Wagner A. R. (1972). Negative patterning in classical conditioning: summation of response tendencies to isolable and configural components. *Psychon. Sci.* 27 299–301 10.3758/BF03328970

[B56] Woocher F. D. Glass A. L. Holyoak K. J. (1978). Positional discriminability in linear orderings. *Mem. Cogn.* 6 165–173 10.3758/BF03197442

[B57] Wright A. A. (1997). Concept learning and learning strategies. *Psychol. Sci.* 8 119–123 10.1111/j.1467-9280.1997.tb00693.x

[B58] Wright A. A. Sands S. F. (1981). A model of detection and decision processes during matching to sample by pigeons: performance with 88 different wavelengths in delayed and simultaneous matching tasks. *J. Exp. Psychol. Anim. Behav. Process.* 7 191–216 10.1037/0097-7403.7.3.191

[B59] Wynne C. D. (1997). Pigeon transitive inference: tests of simple accounts of a complex performance. *Behav. Processes* 39 95–112 10.1016/S0376-6357(96)00048-4 24896713

[B60] Wynne C. D. L. (1995). Reinforcement accounts for transitive inference performance. *Anim. Learn. Behav.* 23 207–217 10.3758/BF03199936

[B61] Wynne C. D. L. (1998). “A minimal model of transitive inference,” in *Models of Action: Mechanisms for Adaptive Behavior* eds Wynne C. D. L. Staddon J. E. R. (Mahwah, NJ: Erlbaum) 269–307

[B62] Yin H. Barnet R. C. Miller R. R. (1994). Trial spacing and trial distribution effects in Pavlovian conditioning: contributions of a comparator mechanism. *J. Exp. Psychol. Anim. Behav. Process.* 20 123–134 10.1037/0097-7403.20.2.123 8189183

[B63] Zeaman D. House B. J. (1962). Approach and avoidance in the discrimination learning of retardates. *Child Dev.* 33 355–372 10.2307/1126449 14009980

[B64] Zentall T. R. (2004). Pavlovian processes in simultaneous discriminations. *Int. J. Comp. Psychol.* 17 185–202

[B65] Zentall T. R. Sherburne L. M. (1994). Transfer of value from S+ to S- in a simultaneous discrimination. *J. Exp. Psychol. Anim. Behav. Process.* 20 176–183 10.1037/0097-7403.20.2.176 8189186

[B66] Zentall T. R. Sherburne L. M. Roper K. L. Kraemer P. J. (1996). Value transfer in a simultaneous discrimination appears to result from within-event pavlovian conditioning. *J. Exp. Psychol. Anim. Behav. Process.* 22 68–75 10.1037/0097-7403.22.1.68 8568497

